# A pilot stratified cluster randomized trial of school-based e-cigarette and other tobacco product use prevention for Pacific Island youths: An evaluation of the Fuetsan Manhoben curriculum

**DOI:** 10.18332/tid/218296

**Published:** 2026-03-24

**Authors:** Francis Dalisay, Scott K. Okamoto, Yoshito Kawabata, Thaddeus A. Herzog, Pallav Pokhrel

**Affiliations:** 1 College of Journalism and Communications, STEM Translational Communication Center, University of Florida, Gainesville, United States; 2 Population Sciences in the Pacific Program, University of Hawai’i Cancer Center, Honolulu, United States; 3 Social and Behavioral Sciences Division, College of Liberal Arts and Social Sciences, University of Guam, Mangilao, Guam, United States

**Keywords:** e-cigarette, adolescents, school-based, curriculum, pacific islander

## Abstract

**INTRODUCTION:**

E-cigarette use among young adolescents in Guam, and potentially other regions of the US-Affiliated Pacific Islands (USAPI), is markedly higher than among similarly aged adolescents in the general US population. The objective was to test the pilot version of a school-based e-cigarette and other tobacco product curriculum, named *Fuetsan Manhoben*, on outcomes related to e-cigarette use among Guam middle school students.

**METHODS:**

The intervention involved 4 video-based, culturally tailored lessons delivered in the classroom by health educators. A stratified cluster-randomized design was used to assign 8 public middle schools to either the intervention or the control condition. Six classrooms in each school participated in the study. Participants (n=269) in the intervention condition received the 4-lesson curriculum, whereas participants (n=269) in the control condition did not receive any intervention. Participants in both conditions provided data at pretest, post-test (4 weeks later), and at follow-up at 3 months. The current analyses pertain only to e-cigarette-related outcomes. Wald tests were utilized to determine the statistical significance of fixed effects within the negative binomial and logistic regression frameworks.

**RESULTS:**

At immediate post-test, the intervention did not have statistically significant effects on openness to using e-cigarettes (incidence rate ratio, IRR=0.79; 95% CI: 0.42–1.49, p>0.05), e-cigarette use initiation (OR=O.96; 95% CI: 0.42–2.19, p>0.05), or past-30-day e-cigarette use (IRR=0.90; 95% CI: 0.77–1.06, p>0.05). At follow-up at 3 months, the intervention also did not have statistically significant effects on e-cigarette use initiation (OR=0.70; 95% CI: 0.34–1.45, p>0.05) or past-30-day e-cigarette use (IRR=0.94; 95% CI: 0.82–1.08, p>0.05). However, findings indicated that the intervention reduced openness to using e-cigarettes at follow-up at 3 months (IRR=0.49; 95% CI: 0.28–0.86, p<0.01). That is, being assigned to the intervention condition was associated with 51% decrease in openness to using e-cigarettes at follow-up at 3 months.

**CONCLUSIONS:**

Four lessons delivered over 4 weeks may not produce immediate short-term preventive effects, except potentially reducing e-cigarette use susceptibility at follow-up at 3 months. Further research is warranted to examine whether expanding the content and duration of the curriculum may result in stronger, more durable effects. Also, future studies should examine whether increasing the follow-up to 6–12 months might capture delayed preventive effects that were not detectable within the shortened, follow-up at 3 months in the current study.

**CLINICAL TRIAL REGISTRATION:**

The study is registered on the official website of ClinicalTrials.gov

**IDENTIFIER:**

ID NCT05037656

## INTRODUCTION

The prevalence of e-cigarette use among middle-school-aged adolescents in the US-Affiliated Pacific Islands (USAPI) is markedly higher than among adolescents of similar age in the US general population^1^. The USAPI consists of Guam, American Samoa, and the Commonwealth of the Northern Mariana Islands (CNMI), which are US territories, and three independent nations in free association with the US – the Republic of Palau, the Federated States of Micronesia (FSM), and the Republic of the Marshall Islands. People of the USAPI face a high burden of cancer and cardiovascular diseases^2,3^. Research increasingly suggests that e-cigarette use is associated with several respiratory disorders independent of cigarette smoking^4,5^.

Guam is the largest and most populous US-Affiliated Pacific Island. The most recently available data indicate that the prevalence of e-cigarette use among middle-school-aged adolescents in Guam is five times higher than among their peers in the US as a whole^1^. Past research shows that Guam youth often begin experimenting with tobacco products during adolescence^1^. Previous research has also found that Guam adolescents are exposed to e-cigarette use offers from peers and family members (cousins, parents, uncles), and that offers for e-cigarettes are most likely to occur in schools than any other location^6^. Furthermore, recent studies show that being able to resist peer influences^7^ and being knowledgeable about the risks involved in using substances predicted lower rates of substance use among Guam youths^1^. These findings underscore the need for interventions that address the social context of e-cigarette offers, equipping Guam youth with the tools and knowledge to resist social pressures to use e-cigarettes. However, prevention efforts targeting initiation in this population remain limited.

### Fuetsan Manhoben school-based curriculum

The pilot version of *Fuetsan Manhoben* (FM, ‘The Power Within Youth’ or ‘The Force Within Youth’ in the Chamorro language) is a 4-lesson, school-based curriculum that represents initial steps in the development of an evidence-based prevention program to meet the urgent need to prevent e-cigarette use among youths in Guam and the USAPI. The curriculum was modeled after and deeply adapted from *Ho‘ouna Pono*^8^, which is a 9-lesson school-based substance use prevention curriculum that has been tested among rural Hawaiian youths and has been found to prevent e-cigarette use^9^. Similar to *Ho‘ouna Pono*, the conceptual framework guiding the current version of *Fuetsan Manhoben* is rooted in the social influence approach of adolescent substance use prevention^10^. Decades of research consistently show that social influences play a central role in shaping adolescent substance use initiation, maintenance, and progression^11,12^.

Several past evidence-based adolescent substance use prevention programs, including school-based programs, have centered around training adolescents in the skills needed to resist social influences that promote substance use^13^. Hence, the core content of the pilot version of *Fuetsan Manhoben* focuses on training middle school students in the skills to resist social influences. Similar to other parts of the USAPI, the core cultural orientation of the majority of Guam’s population is collectivist in nature^14,15^. That is, the cultures of the people of Guam value extended families and familial hierarchies, and encourage in-group conformity and non-confrontation^11-13^. Prevention efforts are therefore most effective when they acknowledge the role of relational decision-making processes and community values.

The FM curriculum was developed through extensive formative research, including multiple focus groups with middle school students on Guam^6,16^. The results of the focus groups helped to delineate specific scenarios in which youths were likely to experience direct and indirect pressures from peers, siblings, and cousins to use e-cigarettes^16^. The formative research also identified scenarios where adults interacted with youths in ways that encouraged tobacco and other substance use^16^. Importantly, this research identified different resistance strategies that youth tended to use to deal with adverse social influences they encountered at home and school, including culturally appropriate non-confrontational strategies^15^.

The present study’s objectives were to pilot-test the feasibility of implementing the curriculum in the classroom setting and obtain preliminary estimates of the short-term effects of the curriculum on e-cigarette-related outcomes. Specifically, we examined the effects of the curriculum on reducing e-cigarette use initiation, current (past-30-day) use, and openness to use e-cigarettes in the future at immediate post-test (i.e. 4 weeks after baseline) and follow-up at 3 months (i.e. 3 months after post-test). These constituted the study’s primary outcomes. The effects of FM on smoking combustible cigarettes and betel nut use-related outcomes were reported in a recent study^17^.

## METHODS

### Study design

The pilot stratified cluster randomized trial (registered as a clinical trial, ClinicalTrials.gov ID: NCT05037656 was conducted between September 2022 through May 2023 and approved by the Institutional Review Board (IRB) at the University of Guam (Approval number: CHRS #22-69; Date: 22 April 2022). Further approval to conduct this study was also granted by the Guam Department of Education (GDOE) and by the principals of each participating public middle school. Parental consent and student assent were obtained before student participation. Eight public middle schools in Guam all participated in the study.

**Figure 1:**
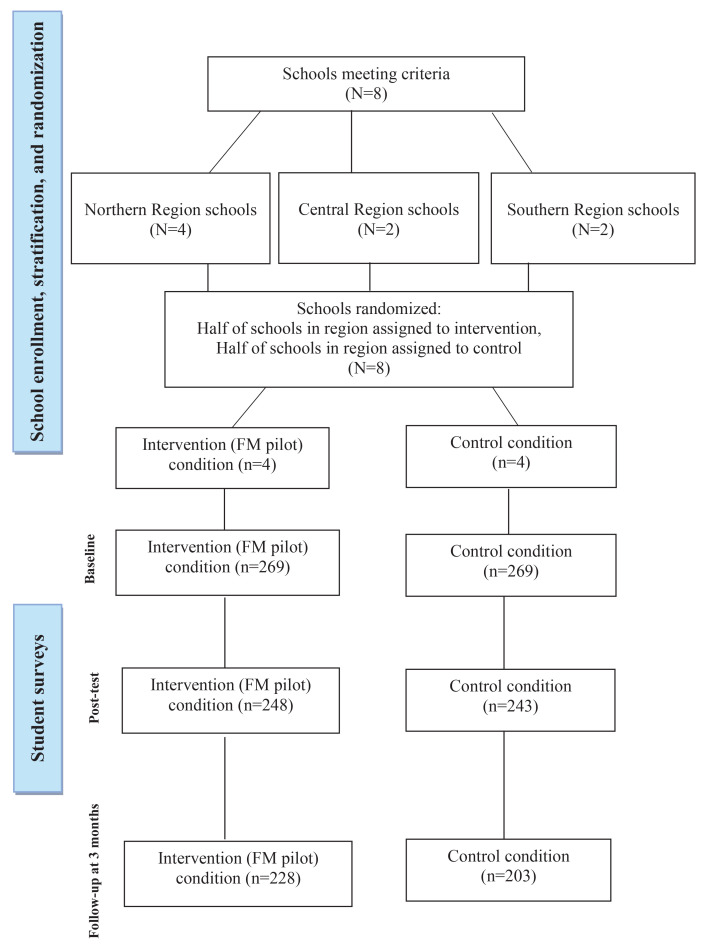
CONSORT diagram showing the flow of participants through each stage of the Fuetsan Manhoben pilot efficacy trial in Guam, 2022–2023 (N=538)

Figure 1 presents the CONSORT diagram outlining the study design and participant flow. As illustrated, the study included both a control group (n=269) and an intervention group (n=269). The control group did not receive the FM curriculum and instead continued with the standard health education curriculum (treatment as usual). In contrast, the intervention group participated in the four FM curriculum lessons (see Table 1 for brief descriptions of each lesson). Data were collected across both conditions at pretest, immediate post-test (i.e. 4 weeks after pretest), and follow-up at 3 months after post-test, using self-administered, self-report survey questionnaires.

**Table 1:** Lessons, videos, and primary content areas of the Fuetsan Manhoben pilot stratified cluster randomized trial in Guam, conducted in 2022–2023 (N=538)

*Lesson*	*Title*	*Video*	*Primary content areas*
1	Introduction to *Fuetsan Manhoben*	‘Introduction’ ‘The Bathroom’	Risk assessment Concepts and terms regarding harms and risks of tobacco product use Health consequences of use
2	Refuse and Explain Yourself	‘The Party’	Cultural values Refusing and explaining
3	Avoid and Stay in Control	‘The Bus’	Avoidance strategies Staying in control through asking questions, brainstorming options, choosing the best option, and doing it
4	Help Yourself and Others	‘The Gym’	Confronting, intervening, negotiating, redirecting, and resisting Final wrap-up summary and pledge

### Participating schools and students

We employed a stratified cluster randomized design in which public middle schools (n=8) were first stratified by geographical region – north, central, and south Guam. The northern region had four public middle schools, while the central region and southern regions each had two middle schools. Within each region, half of the schools were randomly assigned to the intervention condition and the remaining half to the control condition (for example, in the northern region, two schools were allocated to the intervention group and two to the control group). Six classrooms from each school were selected to participate, with an average class size of approximately 15 to 25 students.

### Intervention

The *Fuetsan Manhoben* intervention was delivered in the classroom during regular school hours by trained health educators. Table 1 lists the Fuetsan Manhoben lessons, videos, and primary content areas. The pilot curriculum included four lessons centered around five video vignettes that were developed by the formative research^17^. One video introduced the FM curriculum, while the four additional video vignettes accompanying each lesson portrayed scenarios in which Guam youth were offered tobacco and demonstrated two to three different strategies for resisting these offers.

All four lessons were delivered over a four-week period, with one lesson conducted each week. Each lesson lasted approximately one hour. All lessons followed a standardized structure: 1) each session began with an overview of the lesson topics and an icebreaker activity; 2) next, a video vignette was presented, followed by a short discussion highlighting the cultural values and resistance strategies portrayed; 3) a vocabulary activity then introduced key concepts and terminology for the lesson; 4) the material was then reinforced through one to two interactive activities; 5) finally, the session concluded with a wrap-up activity.

To ensure fidelity throughout the study, each research assistant responsible for delivering the FM curriculum attended a five-hour, in-person training session led by the program developers two weeks before implementation. The training covered all FM lessons, videos, and accompanying handouts, and included role-playing of the interactive activities. During the implementation phase, a co-principal investigator also observed lessons to help monitor and maintain intervention fidelity.

### Data collection process

Data were collected via in-class surveys at three time points: baseline, post-test, and follow-up at 3 months. Both intervention and control groups completed the baseline survey, with intervention participants completing it within three days prior to their first lesson. The post-test occurred approximately four weeks later, shortly after intervention participants finished their final lesson, and the follow-up survey was administered three months afterward. Surveys across waves were linked using participant ID numbers, with the master file stored separately from the raw data. The 80-item survey assessed demographics, intentions to use tobacco and betel nut, and relevant risk and protective factors. Surveys were self-administered under the supervision of research assistants. To maintain confidentiality, no identifying information was collected. Although research assistants were aware of group assignments, potential bias was minimized by using paper-based surveys with standardized instructions. Survey completion took roughly 30 minutes, and participants received a $15 gift card for their time.

### Measures

*E-cigarette use*

Lifetime (ever) use was assessed with a single item: ‘Have you ever in your lifetime used electronic cigarettes (i.e. vape)?’. Response options were ‘Yes’ or ‘No’). To assess past-30-day use, participants were asked: ‘In the last month (past 30 days), on how many days did you use an e-cigarette or a similar vaping device?’. Responses were recorded on a 7-point scale, ranging from ‘0 days’ to ‘daily’. These measures aligned with national surveys to assess tobacco product use^18^.

*Openness to using e-cigarettes*

Four items widely used in the literature to assess e-cigarette use susceptibility among adolescents were used to assess e-cigarette use intentions^19^. Each of the four items (e.g. ‘Do you think you will use an e-cigarette/vape soon?’) was measured on a 5-point Likert scale (0=definitely not, 4=definitely yes). For analysis purposes, responses to each of the four items were dichotomized such that the ‘definitely not’ response was assigned a value of 0 and any other response was assigned a value of 1. An index of openness to using e-cigarettes was created by summing up the dichotomized responses across the four items.

*Demographic variables and potential covariates*

Demographic variables, included as covariates in our regression models described below, included age (years), biological sex (male or female), ethnicity (Chamorro, Filipino, Chuukese, and Other), and household crowdedness (i.e. number of rooms reported in the home divided by number of people living in the home), a proxy for socioeconomic status used in youth surveys^18^.

### Statistical analysis

Statistical analyses were conducted in SAS (Version 9.4). Wald tests were used to assess the statistical significance of fixed effects within the negative binomial and logistic regression frameworks. The analyses focused on the following three outcomes at the immediate post-test and at follow-up at 3 months: e-cigarette use initiation, openness to using e-cigarettes, and past-30-day e-cigarette use frequency. The main predictor was condition – control (0) versus intervention (1). The covariates in our models (Table 2) included age (reported as a mean score with standard deviation), biological sex (reported as % ), ethnicity (reported as % ), and household crowdedness (i.e. number of rooms reported in the home divided by number of people living in the home, reported as a mean score with standard deviation )^20^. E-cigarette use initiation was tested among youths who reported never having used an e-cigarette at baseline (for initiation at immediate post-test) and at baseline and immediate post-test (for initiation at follow-up at 3 months).

**Table 2:** Baseline characteristics for control and intervention conditions of the Fuetsan Manhoben pilot stratified cluster randomized trial in Guam, conducted in 2022–2023 (N=538)

*Characteristics*	*All* *(N=538)* *%*	*Control* *(N=269)* *%*	*Intervention* *(N=269)* *%*	p*
**Age** (years), mean (SD) (range: 10–15)	12.4 (0.95)	12.4 (0.91)	12.5 (0.98)	0.09
**Sex**				
Boys	52	52	52	0.93
Girls	48	48	48	
**Ethnicity**				**0.05**
Chamorro	35	32	38	
Filipino	24	28	20	
Chuukese	31	30	32	
Other	10	10	10	
**Household crowdedness^#^**, mean (SD) (range: 0.29–7.0)	1.8 (0.78)	1.8 (0.80)	1.7 (0.77)	0.67
**Smoking/use**				
Open to e-cigarette use in the future	48	48	49	0.79
Lifetime e-cigarette use	29	29	28	0.73
Lifetime cigarette use	6	6	6	0.73
Past-30-day e-cigarette use	20	20	21	0.12
Past-30-day cigarette smoking	2	2	2	0.26

# Calculated as the number of rooms reported in the home divided by number of people living in the home. Range represents the range in the index of household crowdedness.

* p-value for comparisons between Control and Intervention groups. Independent-samples t-tests were used for continuous variables, where means and standard deviations were compared. Chi-squared tests were used for categorical variables, where percentages (%) are compared.

Because participants were nested within schools, multilevel logistic regression (PROC GLIMMIX) was used to test the effects of the intervention on e-cigarette use initiation. Because of overdispersion in the distributions of outcome variables, negative binomial regression was used to test the effects of the intervention on openness to using e-cigarettes and past-30-day e-cigarette use outcomes at the immediate post-test and at follow-up at 3 months. Openness to using e-cigarettes as an outcome was tested only among participants who reported not being open to using e-cigarettes in the future at baseline (i.e. those who scored 0 on the dichotomized index of openness to using e-cigarettes). For the past-30-day e-cigarette use outcome, the regression model accounted for the baseline past-30-day e-cigarette use. Incidence rate ratios (IRRs) and odds ratios (ORs) were estimated with 95% confidence intervals. Statistical significance for all analyses was evaluated using a threshold of p<0.05.

## RESULTS

### Participant characteristics

As shown on the CONSORT diagram in Figure 1, of the 538 baseline participants, 491 youths (n=248 intervention, n=243 control) participated in the immediate post-test (91% retention), and 431 youths (n=228 intervention, n=203 control) participated in the follow-up at 3 months (80% retention). Both at the immediate post-test and the follow-up at 3 months, those lost to follow-up did not differ significantly from those retained in the study on any of the study measures at baseline. The number of participants retained in each condition over time was >80 per group specified by our initial power analysis.

Attrition analyses were conducted to examine whether participants lost to follow-up at the immediate post-test or follow-up at 3 months differed from those retained in the study on baseline characteristics. Baseline continuous variables were compared using independent-samples t-tests, and categorical variables were compared using chi-squared tests. No statistically significant differences were observed. However, as shown in Table 2, there was a marginally statistically significant difference based on ethnicity (p=0.05), with the intervention and control conditions representing greater numbers of Chamorro and Chuukese youths, respectively.

Table 2 shows the baseline characteristics of participants of the total sample across the intervention and control conditions. At baseline, the mean age of participants was 12.4 (SD=0.95) years, 52% were male (boys), 35% were Chamorro, 24% were Filipino, 31% were Chuukese, and 10% Other. Mean household crowdedness was 1.8 (SD=0.78). For reference, according to the World Health Organization, severe crowding occurs if there are more than 1.5 persons per room^21^; therefore, the mean value of household crowdedness among our sample indicates that, on average, our study’s participants resided in severely overcrowded homes, which correlates with a lower SES background^20^.

### Intervention effects

*Immediate post-test*

Table 3 shows the results of the regression analyses. The intervention was not found to have statistically significant effects on openness to using e-cigarettes (IRR=0.79; 95% CI: 0.421.49, p>0.05), e-cigarette use initiation (OR=0.96; 95% CI: 0.42–2.19, p>0.05), or past-30-day e-cigarette use (IRR=0.90; 95% CI: 0.77–1.06, p>0.05) at the immediate post-test.

**Table 3:** Results of the Fuetsan Manhoben pilot, at immediate post-test, stratified cluster randomized trial conducted in Guam, 2022–2023 (N=538)

*Variable*	*Openness to use^a^*	*p*	*Initiation^b^*	*p*	*Past-30-day use^a^*	*p*
	*Incidence rate ratio* *(95% CI)*		*OR* *(95% CI)*		*Incidence rate ratio* *(95% CI)*	
Condition (Control=0, Intervention=1)	0.79 (0.42–1.49)	0.47	0.96 (0.42–2.19)	0.92	0.90 (0.77–1.06)	0.21
Age (years)	0.98 (0.70–1.37)	0.92	1.03 (0.74–1.44)	0.86	1.09 (0.99–1.21)	0.07
Sex (Boys=0, Girls=1)	1.63 (0.85–2.67)	0.14	1.31 (0.69–2.49)	0.41	1.06 (0.87–1.29)	0.55
Filipino^c^	0.31 (0.14–0.71)**	0.006	0.27 (0.09–0.83)*	0.02	1.02 (0.84–1.24)	0.84
Chuukese^c^	1.26 (0.51–3.10)	0.62	1.08 (0.44–2.66)	0.87	1.19 (1.14–1.23)***	<0.0001
Other ethnicity^c^	0.50 (0.14–1.79)	0.29	0.96 (0.30–3.04)	0.94	0.88 (0.25–3.14)	0.84
Baseline household crowdedness	1.30 (0.83–2.04)	0.26	1.17 (0.82–1.66)	0.38	1.08 (0.93–1.27)	0.32
Baseline past-30-day e-cigarette use					1.26 (1.19–1.33)***	<0.0001

a Results are based on negative binomial regression.

b Results are based on logistic regression.

c Ethnicities were dummy-coded with reference to Chamorro.

* p<0.05,

** p<0.01,

*** p<0.001.

*Follow-up at 3 months*

As Table 4 shows, at follow-up at 3 months, the intervention had no statistically significant effects on e-cigarette use initiation (OR=0.70; 95% CI: 0.34–1.45, p>0.05) or past-30-day e-cigarette use (IRR=0.94; 95% CI: 0.82–1.08, p>0.05). However, we found a statistically significant effect of the intervention on reduction in openness to use e-cigarettes at 3-month follow-up (IRR=0.49; 95% CI: 0.28–0.86, p<0.01). That is, being assigned to the intervention condition was associated with 51% decrease in openness to using e-cigarettes at follow-up at 3 months.

**Table 4:** Results of the Fuetsan Manhoben pilot, at 3 months follow-up, stratified cluster randomized trial conducted in Guam, 2022–2023 (N=538)

*Variables*	*Openness to use^a^*	*p*	*Initiation^b^*	*p*	*Past-30-day use^a^*	*p*
	*Incidence rate ratio* *(95% CI)*		*OR* *(95% CI)*		*Incidence rate ratio* *(95% CI)*	
Condition (Control=0, Intervention=1)	0.49 (0.28–0.86)*	0.01	0.70 (0.34–1.45)	0.33	0.94 (0.82–1.08)	0.39
Age (years)	0.96 (0.73–1.26)	0.79	1.34 (0.90–2.0)	0.15	1.08 (1.0–1.17)*	0.04
Sex (Boys=0, Girls=1)	1.22 (0.76–1.96)	0.46	1.20 (0.58–2.50)	0.63	1.03 (0.86–1.23)	0.74
Filipino^c^	0.45 (0.30–0.68)*	0.02	0.70 (0.27–1.78)	0.46	1.01 (0.83–1.23)	0.92
Chuukese^c^	1.07 (0.72–1.59)	0.90	1.17 (0.43–3.16)	0.76	1.11 (0.89–1.38)	0.36
Other ethicity^c^	0.99 (0.43–2.30)	0.92	1.17 (0.35–3.91)	0.79	0.91 (0.75–1.10)	0.37
Baseline household crowdedness	1.30 (0.79–2.12)	0.39	1.31 (0.91–1.90)	0.16	1.09 (0.82–1.45)	0.52
Baseline past-30-day e-cigarette use					1.25 (1.15–1.36)***	<0.0001

a Results are based on negative binomial regression.

b Results are based on logistic regression.

c Ethnicities were dummy-coded with reference to Chamorro.

* p<0.05,

** p<0.01,

*** p<0.001.

### Ancillary analyses: role of demographic variables

Filipino ethnicity was found to be generally protective against e-cigarette initiation at immediate post-test (OR=0.27; 95% CI: 0.09–0.83, p<0.05), and openness to using e-cigarettes at both immediate post-test (IRR=0.31; 95% CI: 0.14–0.71, p<0.01) and follow-up at 3 months (IRR=0.45; 95% CI: 0.30–0.68, p<0.01). Older age was associated with increased past-30-day e-cigarette use at follow-up at 3 months (IRR=1.08; 95% CI: 1.00–1.17, p<0.05).

## DISCUSSION

This study sought to determine the preliminary feasibility and efficacy of the *Fuetsan Manhoben* pilot curriculum on e-cigarette outcomes. The study mostly found null effects at the immediate post-test and at follow-up at 3 months. Specifically, none of the effects on behavioral outcomes was statistically significant. One of the main reasons for the null effects may be the limited number of lessons in the pilot version of the curriculum. Another reason may be the relatively short follow-up duration. The curriculum was modeled after the *Ho‘ouna Pono* curriculum^8^, which has been found to be effective in reducing e-cigarette use among Native Hawaiian youths in Hawai‘i. The *Fuetsan Manhoben* pilot curriculum, culturally tailored for predominantly Pacific Islander youths from Guam and other USAPI, has 4 lessons, and in the present study, its efficacy was assessed over a 3-month period. However, the *Ho‘ouna Pono* program is a 9-lesson drug use prevention curriculum and the reduction in e-cigarette use in response to the *Ho‘ouna Pono* curriculum was observed over a two-year period^9^. Accordingly, the 9-lesson count could be considered optimal for scaling up the FM curriculum. Further, increasing the follow-up length to 6–12 months might capture delayed preventive effects that were undetectable in the shortened follow-up time frame in the current study. Nonetheless, the effect of the *Fuetsan Manhoben* curriculum on reduced openness to using e-cigarettes in the future suggests that, with an increased number of lessons, the curriculum could have significant preventive effects on e-cigarette use behavior over time.

The present study validated previous findings indicating markedly higher prevalence of e-cigarette use among Guam youths^1^. Given that vaping is even more prevalent in other parts of the USAPI (e.g. the Marshall Islands)^2^, the need for vaping prevention programs that are designed for USAPI youths cannot be stressed enough. A school-based curriculum such as *Fuetsan Manhoben* has the potential reach a large number of youths and generate a substantial public health impact across the USAPI. Hence, continued research on the curriculum may be warranted.

### Limitations

There are limitations to this study. As noted, this was a pilot study with few lessons in the curriculum and a relatively short follow-up duration. Second, the study was based in Guam, which is one island within the sprawling territory of the USAPI. The implications of the current findings for the entire USAPI region are uncertain. Third, we focused on public schools only. This may limit generalizing the findings across the entire population of Guam youths, which includes private and home-schooled students. Fourth, because not all schools were included in the study, there is also the possibility that the study may be underpowered to detect significant changes. Fifth, regarding our attrition analysis, we did not conduct a formal MCAR test, and thus, it is possible that data may not have been missing completely at random. Sixth, even after attempting to adjust for confounding variables, there is still a potential for residual confounding to have occurred. Seventh, due to the self-reported questionnaire, we could not rule out recall bias or social desirability. Lastly, the curriculum was delivered by research staff rather than teachers, which may reduce the external validity of the curriculum.

## CONCLUSIONS

The current study is notable for being the first to systematically develop and evaluate a school-based program aimed at preventing vaping for youths in the USAPI. While the findings support the feasibility of implementing a school-based, video-based curriculum aimed at preventing adolescent e-cigarette use in Guam, the intervention had no significant effects on e-cigarette initiation. However, participation in the intervention was associated with decreased openness to using e-cigarettes at follow-up at 3 months. Yet we note that further research is needed to examine whether expanding the content and duration of the curriculum may result in stronger, more durable effects. Also, future studies should examine whether increasing the follow-up to 6–12 months might capture delayed preventive effects that were not detectable within the shortened follow-up at 3 months.

## Data Availability

The data supporting this research are available from the authors on reasonable request.
