# Corrigendum: A pilot stratified cluster randomized trial of school-based e-cigarette and other tobacco product use prevention for Pacific Island youths: An evaluation of the Fuetsan Manhoben curriculum

**DOI:** 10.18332/tid/226270

**Published:** 2026-07-30

**Authors:** Francis Dalisay, Scott K. Okamoto, Yoshito Kawabata, Thaddeus A. Herzog, Pallav Pokhrel

**Affiliations:** 1 College of Journalism and Communications, STEM Translational Communication Center, University of Florida, Gainesville, United States; 2 Population Sciences in the Pacific Program, University of Hawai’i Cancer Center, Honolulu, United States; 3 Social and Behavioral Sciences Division, College of Liberal Arts and Social Sciences, University of Guam, Mangilao, Guam, United States

**Keywords:** e-cigarette, adolescents, school-based, curriculum, pacific islander

Corrigendum on: A pilot stratified cluster randomized trial of school-based e-cigarette and other tobacco product use prevention for Pacific Island youths: An evaluation of the Fuetsan Manhoben curriculum

By Francis Dalisay, Scott K. Okamoto, Yoshito Kawabata, Thaddeus A. Herzog, Pallav Pokhrel

Tobacco Induced Diseases, Volume 24, Issue March, Pages 1–10

Publish date: 24 March 2026

DOI: https://doi.org/10.18332/tid/218296

In the originally published version of the article, an error was made in the Introduction section, second paragraph, sentence 4: The citation 8 in the phrase “Furthermore, recent studies show that being able to resist peer influences7 and being knowledgeable about the risks involved in using substances predicted lower rates of substance use among Guam youths8”, has now changed to citation 1 (“Furthermore, recent studies show that being able to resist peer influences7 and being knowledgeable about the risks involved in using substances predicted lower rates of substance use among Guam youths1) therefore the rest of the citations from 9-16 were updated accordingly through the manuscript and accordingly were corrected the references 9-16 (9 was updated to 8, 10 was updated to 9, 11 was updated to 10, 12 was updated to 11, 13 was updated to 12, 14 was updated to 13, 14 was updated to 13, 15 was updated 14, 16 was updated to 15. Furthermore, the reference listed as #7 is now updated as follows:

Kawabata Y, Dalisay F, Pokhrel P. Resistance to peer influence, smoking friends, cigarette and betel nut use, and gender among Pacific Islander youth. J Ethn Subst Abuse. 2025;24(4):1045-1063. doi:10.1080/15332640.2023.2295933.

Additionally, citation #8 should be replaced with citation #1.

The mentioned changes are corrected also online.

